# Development and Evaluation of Real Time RT-PCR Assays for Detection and Typing of Bluetongue Virus

**DOI:** 10.1371/journal.pone.0163014

**Published:** 2016-09-23

**Authors:** Sushila Maan, Narender Singh Maan, Manjunatha N. Belaganahalli, Abraham C. Potgieter, Vinay Kumar, Kanisht Batra, Isabel M. Wright, Peter D. Kirkland, Peter P. C. Mertens

**Affiliations:** 1 The Pirbright Institute, Pirbright, Surrey, United Kingdom; 2 Deltamune Pty Ltd., Lyttelton, Centurion, South Africa; 3 Department of Biochemistry, Centre for Human Metabolomics, North-West University, Potchefstroom, South Africa; 4 College of Veterinary Sciences, Lala Lajpat Rai University of Veterinary and Animal Sciences, Hisar, Haryana, India; 5 Elizabeth Macarthur Agricultural Institute, Menangle NSW, Narellan, New South Wales, Australia; 6 School of Veterinary Medicine and Science, University of Nottingham, Sutton Bonington Campus, Leicestershire, United Kingdom; Institut Cochin, FRANCE

## Abstract

*Bluetongue virus* is the type species of the genus *Orbivirus*, family *Reoviridae*. Bluetongue viruses (BTV) are transmitted between their vertebrate hosts primarily by biting midges (*Culicoides spp*.) in which they also replicate. Consequently BTV distribution is dependent on the activity, geographic distribution, and seasonal abundance of *Culicoides spp*. The virus can also be transmitted vertically in vertebrate hosts, and some strains/serotypes can be transmitted horizontally in the absence of insect vectors. The BTV genome is composed of ten linear segments of double-stranded (ds) RNA, numbered in order of decreasing size (Seg-1 to Seg-10). Genome segment 2 (Seg-2) encodes outer-capsid protein VP2, the most variable BTV protein and the primary target for neutralising antibodies. Consequently VP2 (and Seg-2) determine the identity of the twenty seven serotypes and two additional putative BTV serotypes that have been recognised so far. Current BTV vaccines are serotype specific and typing of outbreak strains is required in order to deploy appropriate vaccines. We report development and evaluation of multiple ‘TaqMan’ fluorescence-probe based quantitative real-time type-specific RT-PCR assays targeting Seg-2 of the 27+1 BTV types. The assays were evaluated using orbivirus isolates from the ‘Orbivirus Reference Collection’ (ORC) held at The Pirbright Institute. The assays are BTV-type specific and can be used for rapid, sensitive and reliable detection / identification (typing) of BTV RNA from samples of infected blood, tissues, homogenised *Culicoides*, or tissue culture supernatants. None of the assays amplified cDNAs from closely related but heterologous orbiviruses, or from uninfected host animals or cell cultures.

## Introduction

Bluetongue (BT), is a non-contagious, economically important disease of ruminants (particularly sheep, cattle and some deer species), caused by the bluetongue virus (BTV), which is transmitted primarily by adult *Culicoides* midges [1, 2]. The virus can also be transmitted vertically in vertebrate hosts, and some strains/serotypes can be transmitted horizontally in the absence of insect vectors [3]. The clinical signs of infection can include fever, depression, lameness, oedema of the lips, tongue and head, conjunctivitis, coronitis, excessive salivation, nasal discharge, hyperaemia and pain at muco-cutaneous junctions (such as the gums and vulva) and death. In pregnant animals the virus can cross the placenta, sometimes causing teratogenic effects or abortion including canines [4, 5]. Following recovery, animals may show long-lasting secondary effects, including reduced milk yield or reduced weight-gain, severe wool break and temporary infertility. BT has been listed by the World Organisation for Animal Health as an important transboundary animal disease [6].

BTV genome has 10-segments of linear double-stranded (ds) RNA (identified as genome segments 1 to 10 [Seg-1 to Seg-10] in order of decreasing size), most of which (except Seg-8 and 9) encode a single viral protein [7–9]. The genome segments are packaged within a capsid composed of three concentric layers of proteins. The outer-most capsid-layer is composed of 180 copies of protein VP2 and 360 copies of VP5 (encoded by Seg-2 and Seg-6, respectively). There are 27 known serotypes of BTV (and two additional putative serotypes—Peter Mertens—unpublished data) [10–16] the identity of which is determined by the specificity of reactions between the outer capsid proteins, primarily VP2, and neutralising antibodies that are generated by the vertebrate host (Table 1). Full-genome sequence analyses confirm that VP2 / Seg-2 are the most variable of the BTV proteins / genome-segments [17–20] separating isolates into 29 distinct clades that accurately reflect virus serotype [10, 21]. Studies of reassortant viruses and neutralisation escape mutants indicate that sequence variations in VP5 / Seg-6 can also influence the overall specificity of these neutralisation reactions, although to a lesser extent than VP2 [22–24].

**Table 1:** Isolation details of the known serotypes of BTV.

Serotypes	Year	Origin	Species isolated from	References
1	1958	Biggarsberg—South Africa	Sheep	[54]
2	1958	Vryheid—South Africa	Sheep	[54]
3	1944	Cyprus	Sheep	[70]
4	1900	Cape Province—South Africa	Sheep	[71]
5	1953	Machadodorp—South Africa	Sheep	[54]
6	1958	Vryheid—South Africa	Sheep	[54]
7	1955	Utrecht—South Africa	Sheep	[54]
8	1937	Onderstepoort—South Africa	Sheep	[54]
9	1942	Pretoria—South Africa	Sheep	[54]
10	1956	Portugal	Sheep	[54]
11	1944	Beaufort -West—South Africa	Sheep	[54]
12	1941	Beaufort -West—South Africa	Cattle	[54]
13	1959	Transvaal/Natal—South Africa	Unknown	[54]
14	1959	Transvaal/Natal—South Africa	Unknown	[54]
15	1960	Ermelo district—South Africa	Cattle	[54]
16	1959	West Pakistan	Sheep	[72]
17	1979	United States	Unknown	[73]
18	1976	Republic of South Africa (RSA)	Sheep	(Erasmus, Unpublished)
19	1976	RSA	Sheep	(Erasmus, Unpublished)
20	1978	Australia	*Culicoides*	[74]
21	1980	Australia	Cattle	[74]
22	1992	RSA	*Culicoides*	[75]
23	1987	Australia	Cattle	[76]
24	1992	RSA	Sheep	(Erasmus, Unpublished)
25	2007	Toggenburg—Switzerland	Goats	[77]; [78]
26	2010	Kuwait	Sheep	[14]
27	2014	Corsica, France	Goats	[13]
28	2014	Middle-East	Unknown	Nomikou et al—unpublished
29	2013	RSA	Alpaca	[12]

Part of the table was adapted from Wright, I.M., M.V.Sc thesis [12].

The structural proteins of the BTV core particle (VP1, VP3, VP4, VP6 and VP7), as well as the non-structural proteins (NS1 to NS5) are all more highly conserved than VP2 or VP5, reflecting membership of the same *Bluetongue virus* species / serogroup [15, 25, 26]. However, these analyses have also identified sequence variations in each of the BTV genome segments that group the virus isolates from different geographic regions into ‘major’ eastern and western topotypes (containing isolates from South East Asia, India, China or Australia, or from Africa and North or South America, respectively). These topotype variations are also detected as variations in Seg-2 and Seg-6 (encoding the outer capsid proteins) but within the individual BTV serotypes. There is also evidence for a further ‘far-eastern’ topotype containing viruses from China and Australia, as well distinct groups represented by isolates of the recently discovered serotypes BTV-25 and BTV-26 [10, 18, 27, 28].

Prevention and control of bluetongue relies on preventing the initial introduction of infection into a region or country that contains susceptible hosts and vectors, or on vaccination of susceptible livestock [29], using either live modified viruses, or tissue culture derived and chemically inactivated virus preparations [30–32]. The ruminant host’s response to vaccination is serotype specific and rapid, therefore accurate and reliable serotype identification is an important part of any surveillance and control programme, to support the design and rapid deployment of appropriate vaccines. Diagnostic systems for BTV are also needed to demonstrate absence of the virus in individual animals or animal products (for safe movements/export/import), as well as for declaration of a ‘virus free’ status, after outbreaks in non-endemic countries or zones [6].

The bluetongue viruses were initially identified and distinguished from the other orbiviruses by serological methods, including Agar gel immuno-diffusion (AGID) tests and enzyme-linked immunosorbent assay (ELISA) [33]. However, these methods are slow, labour intensive and require virus isolation and/or access to standard reagents (antibodies and antigens) that may themselves represent a potential biosecurity risk.

BTV RNA can be detected by amplification in conventional or real-time RT-PCR assays. Several of the ‘conserved’ BTV genome segments have been used as targets for conventional RT-PCR assays, including the genes encoding VP1, VP3, VP7, NS1, NS2 and NS3 [34–36]. Similarly, several BTV genome segments have been targeted for real-time RT-PCR assays for detection of BTV RNA, including those encoding VP1 [37, 38]; NS1 [38], NS2 [39], VP6 [40] and NS3 genes [41]. Since none of these proteins determine BTV serotype hence these targets cannot be used to ‘type’ BTV isolates.

Virus neutralisation tests (VNT) and serum neutralisation tests (SNT) are the “gold standard” method for BTV serotyping. However, serological procedures are expensive and time-consuming and are often associated with poor sensitivity. Unlike conventional PCR-based approaches, quantitative real-time PCR chemistries use fully automated detection systems that eliminate the need for post-amplification sample handing, hence displaying very high sensitivities and broad dynamic ranges after optimization particularly for mixed infections.

Highly sensitive and specific molecular ‘typing’ assays, targeting Seg-2 (including conventional, RT-PCR, sequencing and probe-hybridisation methods) have previously been reported for identification of 26 BTV and 9 AHSV serotypes [21, 42, 43]. However, the detection of novel serotypes and further introductions of exotic serotypes and topotypes into new geographic areas, requires a complete set of Seg-2 specific TaqMan assays for accurate, sensitive and specific serotype identification of all of the known BTV types, to help in surveillance as well as the rapid design and implementation of effective vaccination and control programmes. Full-length Seg-2 sequence data from multiple isolates of individual types, including strains belonging to both eastern and western topotypes, were used to design a most complete set of ‘serotype-specific’ primers and probes to detect and identify Seg-2 from all known serotypes of BTV with the exception of putative BTV-28. The specificity of each assay was evaluated using a wide range of BTV isolates from the Orbivirus Reference Collection (ORC) [44] at The Pirbright Institute (TPI).

The real-time RT-PCR assays that are described in this manuscript provide rapid and reliable BTV serotype identification and are suitable for high through put diagnostic systems. They are not invalidated by mixed infections and have been used to identify incursions of multiple BTV types into Europe, India, Australia, Middle East and the USA [10, 11, 45–47].

## Materials and Methods

### Primers and probe design

Seg-2 nucleotide sequences of BTV reference strains that were generated during this study using the previously described method [25] and nucleotide sequences that are already available in the public domain (GenBank) were used to design primers and probes for the detection and typing of BTV RNA (S1 Table). For this the Seg-2 sequences were aligned and analysed collectively and separately for each of the 27+1 BTV serotypes using MEGA v. 6 [48]. Unique regions in Seg-2 were identified for each serotype, as targets for the primers and probes (Table 2). The primers and probes for each serotype specific assay, were also checked using the available sequence data to ensure no cross-reactions with the genome segments of related BTV serotypes or any of the 29 heterologous *Orbivirus* species [25, 44, 49–52]. Probes were labelled at their 5’ and 3’ ends with 6-carboxyfluorescein (6-FAM) and Black Hole Quencher-1 (BHQ-1) respectively. All oligonucleotides were synthesised by Eurogentec, UK. Primers were PAGE purified and probes were HPLC purified.

**Table 2:** List of primers and probes for BTV type-specific assays.

Oligo Name	Oligo Sequence (5'-3')	Pd Size (bp) eastern (e) or western (w)
**BTV-1 (eastern + western strains)**		
BTV-1/2604-2627P(w)	CCGATCACACATCCGAACAAATGC	78 w
BTV-1/1632-1662P(e)	CAACGACRGAAYGATGAYCCRATGGTGAAAC	112e
BTV-1/2575-2597F(w)	GTATTTCTGAYGGTATTGTYTGG	
BTV-1/1599-1623F(e)	GCYAAATTRCGAATCAARCATRGYG	
BTV-1/2653-2633R(w)	TCATCAGATACCTCGATCGCT	
BTV-1/1711-1689R(e)	GTTARCCTCTGCAAYACAATAGG	
**BTV-2 (eastern + western strains)**		
BTV-2/1455-1428P(w)	CATTCCATCCACCATCTATAATTTCCCC	127w
BTV-2/116-139P(e)	CCAAGATGGCCGACATGACGTATC	110e
BTV-2/1401-1421F(w)	GATGAYRYAARTAYTCTGAG	
BTV-2/60-81F(e)	GAGCATTTGTTGAAARGTTATG	
BTV-2/1528-1503R(w)	GYATCYYTTTCGAARTCRATTGTRAG	
BTV-2/170-148R(e)	GATATCRAAYGCGTACATYTCTG	
**BTV-3 (western strains)**		
BTV-3/S2/656-688Pw	CYCCRCAGTTTCAYACAATACAGAGGAACCATC	99w
BTV-3/S2/619-640Fw	GARCGGTTRTCRACGGAWGARG	
BTV-3/S2/718-694Rw	TATCRTAAGCGTTATCTCCTARCYG	
**BTV-4 (western strains)**		
BTV-4/S2/2502-2529P2	TACCTGTTGTGACRTCCAAGTTGGACAC	87w
BTV-4/S2/2470-2488F2	GAACACGAAGATATCGCAG	
BTV-4/S2/2557-2532R2	GCATARAGAAGCTARATGTATCTTCA	
**BTV-5 (western strains)**		
BTV-5/S2/36-61Pw	CCGATWTTKCGRTCGAGCCAAGTTCC	93w
BTV-5/S2/08-26Fw	GCTTCTCAGGATGGATGAG	
BTV-5/S2/101-79Rw	CARRTCRAYCTTAAYRTCRTAYC	
**BTV-6 (western strains)**		
BTV-6/2086-2061P	CACCTTGAYTCATCCACACTACGAAC	111w
BTV-6/2001-2023F	GTCGATGTYACACAGTTGATCGT	
BTV-6/2112-2090R	TAGCACGTCTAATCGTTTCTATG	
**BTV-7 (western strains)**		
BTV-7/S2/1635-1660Pw	CCACAATCTAGACCCGGCAATATCGC	96w
BTV-7/S2/1608-1631Fw	AGTATGTGAGACGTCAATCTCAGA	
BTV-7/S2/1704-1682Rw	GTCTAATAGGTCCGCAGCTTTAG	
**BTV-8 (western strains)**		
BTV-8/132-106P(w)	CGGGCTCATCACCTTCCTCTTCAACAC	87w
BTV-8/72-93F(w)	GATGGRTATGATTACATCATTG	
BTV-8/159-138R(w)	GAATTYCTGTYACATCGTGTCG	
**BTV-9 (eastern + western strains)**		
BTV-9/1703-1727P(w)	CTTATATGACACTCGCCCTGCCATC	106w
BTV-9/1735-1762P(e)	CAACCCTATCAATGAGACAACGCCAGAC	97e
BTV-9/1673-1694F(w)	GGTTATGCTTCAATTACGAACG	
BTV-9/1706-1724F(e)	GTATGATACCAGGCCAGCG	
BTV-9/1779-1756R(w)	GGGTCTTATGTAGGGATGTCTGTG	
BTV-9/1803-1783R(e)	GTTCATTTTGAGGATCATCCA	
**BTV-10 (western strains)**		
BTV-10/S2/1519-1552Pw	YCTTGGYNCGCGYTCTGAATTAGTATTYCCRCCY	107w
BTV-10/S2/1470-1488Fw	TATTRACWACWGAACCAAACCT	
BTV-10/S2/1577-1557Rw	GYGARTTRATCCRTTTGTCAT	
**BTV-11 (western strains)**		
BTV-11/S2/1540-1573Pw	YGTGCTCCCAAGTTATTTCGATCAATGGATCTAC	107w
BTV-11/S2/1510-1530Fw	GGATGCGYAYYTGAATATTAG	
BTV-11/S2/1617-1596Rw	ATCTCTCCATGAGTTATTCGCA	
**BTV-12 (western strains)**		
BTV-12/S2/1101-1077Pw	CTCCACCATATGCGCCAACGATAGC	137w
BTV-12/S2/999-1019Fw	ATACAATTCAGGCTATCCRGA	
BTV-12/S2/1136-1116Rw	CAATGATYGTTCCTCGTAAGC	
**BTV-13 (western strains)**		
BTV-13/S2/1206-1175Pw	CTTATATCCCTCACGTACGCTCCAYTCATACC	78w
BTV-13/S2/1147-1169Fw	GGTGACGTYTATTATAAATTGCG	
BTV-13/S2/1225-1207Rw	GGCGATCCARATCYCGWGG	
**BTV-14 (western strains)**		
BTV-14/S2/2663-2683Pw	CCGGCTTCGCGCGAGRTTYCC	142w
BTV-14/S2/2616-2636Fw	GCCATTGARTTTTCTGAYGAYAG	
BTV-14/S2/2758-2734Rw	TCWGTATAYGCCTTAACYGCTCT	
**BTV-15 (western strains)**		
BTV-15/S2/130-105Pw	CCCTCCCGATAAAGCGACCATATTCC	148w
BTV-15/S2/29-47Fw	CCTGTGAGCGTGATCGAAC	
BTV-15/S2/177-156Rw	CTTACACCTATGTTTCGCACTC	
**BTV-16 (eastern + western strains)**		
BTV-16/1291-1264P(W)	CCTTCGTTGCTGGCTCTCCCTCTAGATC	116w
BTV-16/1291-1264P(e)	CCTTCGTTGCRGGCTCTCCTTCTAAGTC	127e
BTV-16/1221-1243F(W)	GCGAGAGCAAGAGAAGTATATCG	
BTV-16/1193-1213F(e)	GACCTGAATATAAACCGCGAG	
BTV-16/1337-1319R(W)	GATGTTCGATACGTCTGGG	
BTV-16/1320-1297R(e)	ATTAATCAATTCGTACTCCCAGTG	
**BTV-17 (western strains)**		
BTV-17/S2/2224-2254Pw	CCTCCCTCTGATGTTCCTTGTTCATGATAAC	137w
BTV-17/S2/2178-2202Fw	TGCTRAAAGAGATCAAATTTGTRCGG	
BTV-17/S2/2315-2295Rw	ACTTGATCGTATCGTCAAACA	
**BTV-18 (western strains)**		
BTV-18/S2/387-414Pw	CATGTACCATCACGGATAAGCCACGCCC	94w
BTV-18/S2/357-381Fw	GATTATCAACCACTTAAGGTCGACG	
BTV-18/S2/451-425Rw	GCTCTCTTTGCGTGTAACCTTACCGTG	
**BTV-19 (western strains)**		
BTV-19/S2/2378-2346Pw	CCAAACCTATTATARTACGCACCRAGCTCAACC	97w
BTV-19/S2/2313-2336Fw	AGTGTTGRTATCRCATAAATTACG	
BTV-19/S2/2410-2379Rw	GGAAAGTYAGATGCGAAATYARRGAAGTCAAT	
**BTV-20 (western strains)**		
BTV-20/S2/1876-1902Pw	CCGTAAAACCGCTTTGATGCTGATGGC	90w
BTV-20/S2/1838-1856Fw	GCAATATGTCCGCATGCTG	
BTV-20/S2/1928-1909Rw	GCTCCGGGCTTAATTTTTCG	
**BTV-21 (eastern strains)**		
BTV-21/S2/1613-1636P	CGCTCAACGTAAAGCAGATGACCC	102e
BTV-21/S2/1584-1603F	GCCAGATTAAAGATAACGCA	
BTV-21/S2/1686-1669R	GTAAATCGATAGGGTCCG	
**BTV-22 (western strains)**		
BTV-22/S2/1101-1077Pw	CTCCACCAGATACGCCACCGATAAC	111w
BTV-22/S2/1013-1032Fw	ATCTCAAGCGGTCAAACAGA	
BTV-22/S2/1124-1148Rw	CCATTTCACAYGCTATTATAGTTCC	
**BTV-23 (eastern strains)**		
BTV-23/S2/92-118P	CGAYGTAAGCACACGYATCGATGAACC	88e
BTV-23/S2/60-81F	GCGGARYTGTTAGATGGCTATG	
BTV-23/S2/148-126R	GGAATTTGWGYRACRTCATGACG	
**BTV-24 (western strains)**		
BTV-24/S2/1944-1973Pw	CATCAGACTTACAYGCACCCGAARATAAAY	115w
BTV-24/S2/1901-1919Fw	GAACTAYGAGAAGCTTAYR	
BTV-24/S2/2016-1994Rw	GCGAAAARTCYYTCATATCTA	
**BTV-25 (western strains)**		
BTV-25/S2/2576-2605P	CCCTCCCAATAACACATCCAGAGAAGTGCC	90w
BTV-25/S2/2554-2571F	TTATCGGACTCGCTCGTT	
BTV-25/S2/2644-2622R	GTGGATTCAACTTATTATCTCCG	
**BTV-26 (eastern strains)**		
BTV-26/S2/1796-1827P1	CGAGAGGACTTCGCTATGCTAACACATTACGC	97e
BTV-26/S2/1752-1771F1	GTTATAGGCAGCAGCAATCT	
BTV-26/S2/1849-1831R1	GCATATATCCCTTTCACCT	
**BTV-27 (western strains)**		
BTV-27/S2/1392-1420P	CAACGGCATGCGTGATGATATTATACGGC	116w
BTV-27/S2/1334-1354F	GCAAATCACAAGAAAAAGAAG	
BTV-27/S2/1450-1423R	GTAACTTCAAGCTTTCCTGGCG	
**BTV-29 (western strains)**		
BTV-29/S2/1541-1572P	CATCTCGATAACCGCAATCACCTAGTGATGCC	140w
BTV-29/S2/1502-1522F	GAAAAGAACATCTTCAACATG	
BTV-29/S2/1642-1615R	GATCTCAGTCGTGGTTACTCT	
**Additional list of primers and probes for BTV type-specific assays**		
**BTV-1 (eastern + western strains)**		
BTV-1/2604-2627P(w)	CCGATCACACATCCGAACAAATGC	78w
BTV-1/1630-1662P(e)r	CASAACGACRGAATGAYGAYCCRATGGTGAAAC	121e
BTV-1/2575-2597F(w)r	GTATTTCTGAYGGTATYGTYTGG	
BTV-1/1590-1615F(e)r	ATGTTTAAYGCYAARTTRCGAATYAA	
BTV-1/2653-2633R(w)r	TCATCAGAYACCTCGATCGCY	
BTV-1/1711-1689R(e)	GTTARCCTCTGCAAYACAATAGG	
**BTV-4 (eastern strains)**		
BTV-4/S2/1454-1430P(e)	CCGCTCTTGATCCCACCCACCTTGA	125e
BTV-4/S2/1379-1400F(e)	TTGTGTAAAGTGGATGAGGAGA	
BTV-4/S2/1504-1477R(e)	GAAGTCTATCGTCAAAAGGTTAGGGGCT	
**BTV-12 (western strains)**		
BTV-12/S2/1101-1077Pw	CTCCACCATATGCGCCARCGATAGC	137w
BTV-12/S2/999-1019Fw	ATACAATYCAGGCYATCMRGA	
BTV-12/S2/1136-1116Rw	CAATGATYGTTCCTCGTAAGC	
**BTV-21 (eastern strains)**		
BTV-21/2582-2601Pe	CCTCCCAATAACGCATCCGC	75e
BTV-21/2562-2579Fe	ACTGAGGGATTAGTTTGG	
BTV-21/2637-2617Re	GRTCATCRCAAATTTCAATSG	

### Virus isolates

A total of 1063 BTV isolates (from ORC) representing all 27+1 of the known BTV serotypes, and strains from different areas of the world, were used for evaluation of the real-time RT-PCR assays (S2 Table) [44]. Other *Orbivirus* species isolates were also used to assess the specificity of the BTV-serotype-specific assays. All of the isolates were grown in BHK-21 clone 13 cells (European Collection of Animal cell Cultures [ECACC– 84100501]), Vero cell monolayers (ECACC– 84113001), or in KC cells derived from *Culicoides sonorensis* [53]. Infected KC cells were harvested 7 days post infection, whereas the infected mammalian cells were harvested when ~80% cytopathic effect (CPE) was observed. In these studies the virus isolates were generated from diagnostic samples (blood and/or spleen or lung), taken from naturally infected animals as part of normal veterinary surveillance in the respective countries. Established reference strains of BTV were also included in these studies [54]. Neither animals were infected nor samples were taken from animals specifically for these studies, hence further ethical approval was not sought.

### RNA isolation

dsRNA was extracted, either from tissue culture supernatant, blood or other clinical samples, using a MagVet^™^ universal purification kit (Kingfisher^™^, Life Technologies), or from BTV infected cells (using Trizol Reagent^®^, Invitrogen, UK) [55]. In the first method, 100 μl of infected-tissue-culture supernatant/blood/clinical sample was added to 250 μl of the lysis buffer (NM1). Total nucleic acid (80 μl) was extracted from this solution using the Kingfisher^™^ platform, with magnetic beads and Kingfisher^™^, automates. Each of the isolates was handled with care to avoid any cross-contamination. Total nucleic acid from uninfected tissue culture supernatants, sheep blood or homogenised *Culicoides* was also extracted using Kingfisher^™^ (Life Technologies) system.

### RT-PCR reactions

RT-PCR reactions were performed as described previously by [56]. Briefly, a fragment of the targeted genome segment was amplified with different primers-probe sets using SuperScript III/ Platinum Taq One-Step qRT-PCR Kit (Invitrogen, UK). RNA samples were always heat denaturated (95°C for 3 min) prior to addition to the reaction mix [37]. Amplification was carried out in MX3005p (Stratagene, UK) using the conditions: 55°C for 30 min, 95°C for 10 min followed by 45 cycles of 95°C for 15 s and 60°C for 1 min. Fluorescence was measured during a 60°C annealing/extension step. Cycle threshold (Ct) values were measured as the point at which the sample fluorescence signal crossed a threshold value (the background level). Negative results (for assays that did not exceed this level of signal) are reported as ‘No Ct’. All precautions were taken to avoid accidental contamination. The composition of the individual optimised assays using either one or two sets of primers and probes is presented in Table 3.

**Table 3:** Composition of reaction mixes for one-step RT-PCR reaction with one set of primers and probe (3a) and two sets of primers and probes (3b).

**Table 3a—One-step RT-PCR reaction mix with one set of primers and probe**	
**Reagent**	**Amount (μl)**
Forward primer (10 pm/μl^-1^)	1
Reverse primer (10 pm/μl^-1^)	1
Probe (5 pm/μl^-1^)	1
MgSO_4_ (50mM)	1
ROX (1/10 dilution)	0.5
^1^Superscript^®^ III RT/Platinum^®^ Taq Mix 2x reaction mix	12.5
Nuclease free water (μl)	5
dsRNA (μl)	3
**Total volume (μl)**	**25**
**Table 3b**^2^**- One-step RT-PCR reaction mix with two sets of primers and probes**	
Forward primer 1 (10 pm/μl^-1^)	1
Reverse primer 1 (10 pm/μl^-1^)	1
Probe 1 (5 pm/μl-1)	0.5
Forward primer 2 (10 pm/μl-1)	1
Reverse primer 2 (10 pm/μl-1)	1
Probe 2 (5 pm/μl-1)	0.5
MgSO_4_ (50mM)	1
ROX (1/10 dilution)	0.5
Superscript^®^ III RT/Platinum^®^ Taq Mix 2x reaction mix	12.5
Nuclease free water (μl)	3
dsRNA (μl)	3
**Total volume (μl)**	**25**

^1^SuperScript III/ Platinum Taq One-Step qRT-PCR Kit (Invitrogen, UK).

^2^The duplex format of the assay is for use with sets of primers and probes for the same serotype (eastern and western strains).

### Evaluation of RT-PCR assays for diagnostic specificity

A panel of (1063) isolates representing BTV serotypes 1 to 24, -26, -27 and -29, from different geographical locations, were obtained from the ORC (S2 Table; [44] and tested in each case in triplicate. The Seg-2 based type-specific assays were tested *in vitro* with both homologous and heterologous serotypes to ensure diagnostic specificity and sensitivity. Isolates for BTV-25, and -28 were not yet available from the ORC for testing *in vitro*, so the primers and probes for BTV-25 were tested against them by sequence comparisons only, while for BTV-28 Seg-2 sequence data was not available for design of primers.

Representative isolates of other *Orbivirus* species, including *African horse sickness* (AHSV), *Epizootic haemorrhagic disease virus* (EHDV), *Equine encephalosis virus* (EEV) and *Peruvian horse sickness virus* (PHSV) (as listed in S2 Table), were used to evaluate and confirm the diagnostic specificity the BTV-specific assays. The typing assays are also used extensively in the European and OIE reference laboratory at Pirbright and by colleagues in laboratories in the Americas, Asia, Europe and Australasia to identify the serotype of novel BTV isolates [10, 46, 47, 57–59] (S2 Table). To ensure that no false positives are caused by cross-reactions with the host-species, total nucleic acid was extracted and tested from uninfected BHK, Vero and KC cell-culture supernatants, sheep and cattle blood and homogenised *C*. *sonorensis*, with uniformly negative results (S2 Table).

### Evaluation of sensitivity and efficiency of the assays

The analytical sensitivity of the Seg-2 type-specific assays for the European serotypes (BTV-1, -2, -4, -6, -8, -9, -11, and -16) were assessed using a dilution series of the quantified dsRNA genome of reference strains of each BTV serotype (as listed in S1 Table). Additionally, this selection of the assays for the European serotypes were also assessed for their sensitivity and efficiency, using a recombinant plasmid DNA having insert sizes ranging from 78–127 bps (Seg-2 specific amplicons for each of the eight European serotypes).

The dsRNA standards were prepared as follows: viral dsRNA (of RSArrrr/01 or other reference strains) was extracted as previously described [55], assessed for any ssRNA remains in 1% agarose gel electrophoresis and the concentration of dsRNA was determined with NanoDrop (Thermo Fisher Scientific, USA). To test analytical sensitivity, a 10-fold dilution series of dsRNA (10^10^ to 10° copies per μl) was made in a sample of RNA extracted from uninfected BHK cells supernatant, then tested in triplicate. The number of dsRNA copies was calculated with the formula: Y = (X/(a x 680)) x 6.022 x 10^23^, where: Y = molecules/μl; X = g/μl dsRNA; a = viral genome length in nucleotides; 680 is the average molecular weight per nucleotide of dsRNA.

To test analytical sensitivity of the Seg-2 type-specific assay using recombinant plasmid DNA, a 10-fold dilution series of plasmid DNA (10^10^ to 10° copies per μl) was made in TE dilution buffer and then tested in triplicate. The number of plasmid DNA copies were calculated with the formula: Y = (X/(a x 660)) x 6.022 x 10^23^, where: Y = molecules/μl; X = g/μl dsDNA; a = plasmid plus insert length in nucleotides; 660 is the average molecular weight per nucleotide of dsDNA.

Viral dsRNA dilution series were used to generate standard curves by linear regression methods, setting Ct values as dependent, and the dsRNA concentrations as independent variables. The slope of the standard curves for the optimised serotype-specific assays, were then used to estimate the efficiency of the individual assays in detection of each of the reference strain. The efficiency was calculated by the formula *E*% = (10^−1/*slope*^−1)×100. Efficiencies of Seg-2 assays were estimated on the basis of standard curves plotting Ct values against corresponding log plasmid copy number per reaction.

## Results

### Design of serotype specific primers—probes for different BTV ‘types’

Sequence data generated for Seg-2 of multiple field, reference and vaccine strains as well as those available publically in GenBank (S1 Table), were compared to select regions unique to each of the virus types for the design of primers and probes that could be used to distinguish and detect each of the 27 + 1 BTV serotypes (Table 2).

The sequences and positions for the serotype-specific primers for BTV serotypes 1–27 and putative BTV-29 are given in Table 2. Seg-2 sequence data for assay design for putative BTV28 was unavailable. In some cases (e.g. BTV-7, -18, -21 and -24) the number of available virus strains, topotypes and / or sequences for each serotype is limited. This imposes limits on the designs of the current assays, as well as on their evaluation and testing, suggesting that in order to maintain their efficiency the re-design of some assays may be necessary, as additional isolates and new topotypes of each serotype become available.

In some cases, for example during testing of the BTV-1 specific assays, some of the eastern topotype strains from Australia (AUS2003/01, AUS2005/02, AUS2009/02) were not detected by the original primers and probes. The type 1 assay was therefore redesigned with a new probe and three out of four primers (Table 2). Similarly the BTV-4, BTV-12 and BTV-21 assays were redesigned to maintain their efficiency for the detection of Indian, Zimbabwean and Brazilian strains (Table 2).

### Evaluation of RT-PCR assays targeting Seg-2, for type-specificity

The RNAs of the 27 monotypic BTV reference strains (BTV-1 to 24, -26, -27 and -29), were tested in triplicate, with ‘typing-assays’ targeting Seg-2 of each BTV serotypes (S2 Table). Due to non-availability of RNA for BTV-25 only sequence based comparison/evaluation was done for this serotype. As the sequence data for BTV-28 was not available, the assay for this serotype could not be designed. In every case amplification was only observed with the homologous assay confirming the specificity of the assays for each serotype (S2 Table). The specificity of the assays was further evaluated using field and/or vaccine strain isolates of each serotype, where possible representing different topotypes collected from diverse geographic locations (from the ORC). Positive signals were only obtained with the homologous serotype (S2 Table).

RT-PCR ‘typing’ results for majority of the isolates tested were subsequently verified by sequencing and phylogenetic analyses of Seg-2 (S2 Table). With some strains the original primers and probes (as mentioned in table 2) showed relatively poor reactivity. These included some of the isolates of BTV-1 from Australia (AUS1996/03; AUS2003/01; AUS2005/02; AUS2009/02) and BTV-4 strains isolated in 2014 from Southern India (IND2014/24), BTV-12 from India (IND2012/01), Brazil (BRA2002/01) and Zimbabwe (ZIM2003/04) and BTV-21 from India (IND2007/09) (S1 Table). The Seg-2 sequence data from these strains showed variations in the primer and/or probe ‘foot-prints’. Additional sets of primers and probes were therefore designed and tested for RNA isolated from blood and cell culture grown samples of these serotypes (Table 2).

Nucleic acid preparations derived from uninfected hosts species (sheep and cattle blood and *C*. *sonorensis*) or uninfected cell culture supernatants (KC cells, BHK cells and Vero cells), or dsRNA from other closely related *Orbivirus* species (EEV, PHSV, EHDV and AHSV) (S2 Table) gave ‘no Ct’ values in any of the type-specific assays. Sequence comparison of Seg-2 sequence data for 29 *Orbivirus* species [44], indicated that neither primers nor probes of the BTV type-specific assays, would bind to, or amplify their RNA.

Some of the BTV strains originating from India, Israel, Turkey, Europe and, Africa, were tested using serotype-specific TaqMan probe based qRT-PCR assays giving positive results for more than one serotype, reflecting the co-circulation of multiple serotypes [44]. For example, The Indian isolate IND2005/06 contains BTV-2 and BTV-9; Indian isolate IND2005/05 contains BTV-1, BTV-10 and BTV-23; Israeli isolate (ISR2010/22) contains BTV-15 and BTV-8; isolate ISR2010/16 contains BTV-4 and BTV-8. Isolates TUR2000/06 and TUR2000/07 from Turkey contain BTV-9 and BTV-16 [44]. These real-time RT-PCR data were confirmed by conventional Seg-2 specific qRT-PCR assay [21] and Seg-2 sequencing of the mixed BTV strains, showing more than one distinctive consensus sequences in each case. Comparison of the sequence data to the Seg-2 dataset of the 29 BTV reference strains, confirmed the real-time RT-PCR typing results.

### Use of RT-PCR assays for typing of BTV isolates from disease outbreaks

The type-specific qRT-PCR assays described here, were used for the primary identification, or confirmation of BTV serotype for multiple (>1000) diagnostic samples, and field isolates of BTV. These type specific qRT-PCR assays were used during August—September 2006 for the identification of BTV-8 in clinical samples from animals in the Maastricht region of northern Europe, and again in 2007 from the UK. Late in 2008/early 2009, BTV-6 was identified in the Netherlands [11], BTV-11 in Belgium [57], BTV-25 in Switzerland [17] and BTV-26 in Kuwait [14], BTV-27 in Corsica [13], and BTV-29 from South Africa [12]. The assays and primers described here are now in routine use in the OIE reference laboratory at The Pirbright Institute, to detect and identify any of the BTV serotypes as listed on the ORC web pages [44] in diagnostic samples. The results of these assays can be (are) followed by full genome sequencing and phylogenetic analysis of representative samples from each outbreak, to confirm serotype, and identify topotype, and genotype (revealing the lineage / origins of each genome segment and identifying reassortant strains [10, 28].

The BTV serotypes involved in outbreaks that occurred in southern India during 2014 were identified using these qRT-PCR methods, including BTV-2, BTV-4 and BTV-12 (Maan et al—unpublished data). BTV-1 (SPA2014/08), and BTV-4 (SPA2014/06, SPA2014/07) were identified in Spain and the BTV-4 outbreak that occurred in South-eastern Europe during 2014 and 2015, were all identified in this way (BUL2014/01 to BUL2014/14; KOS2014/04 to KOS2014/09) (S2 Table). Viruses were also isolated from bovine semen samples collected in 2011 from Brazil. One of the isolates (BRA2011/02), contained BTV-4, BTV-8, BTV-10 and BTV-16, while another isolate (BRA2011/01) contained BTV-4 and BTV-10 (Gasparini et al—in preparation) (S2 Table). This provides the first report of BTV-8, BTV-10 and BTV-16 in Brazil.

Previously exotic serotypes that were identified using these techniques, include: BTV-5 and -24 in India during 2014 (Hemadri et al—unpublished data; [60]; BTV-1, -3, -5, -6, -14, -19, -22 and -24 from the Americas during 2006–2007 [46]; BTV-1, -4, -8 and -16 from Oman; BTV-15 and BTV-24 from Israel [61]; BTV-2, -5, and -7 from Australia (S2 Table; Peter Kirkland—Personal communication).

### Analytical sensitivity and efficiency

RNA of the homologous BTV serotype was detected by all of the ‘typing’ assays. A selection of the assays for the European serotypes (BTV-1, -2, -4, -6, -8, -9, -11, and -16) were tested for their sensitivity and efficiency. Plasmid DNA corresponding to homologous European BTV serotype Seg-2 RNA targets were detected by respective ‘typing’ assays, at all nine dilutions down to 2–11 copies (S3a–S3h and S4 Tables).

Efficiency rates were calculated for the different European serotype typing assays, on the bases of dilutions series of dsRNA, giving values between 95–102% (S4 Table) reflected by a range of slope values (between -3.2 and -3.4). All eight of the Seg-2 assays for European serotypes that were tested against their homologous serotypes showed linearity (*R*^2^ > 0.99) (S4 Table).

## Discussion

Bluetongue virus causes severe and economically important diseases in domesticated and wild animals. Introduction of these viruses into areas which are usually free from the disease having immunologically naïve populations of susceptible host can cause high morbidity and mortality [62]. Even in endemic areas, BTV can cause massive reduction in overall productivity of animals [63]. Apart from emergence of new reassortant strains with novel characteristics, massive genetic and antigenic diversity among different serotypes/types make the prevention and control of BTV very difficult.

To enable the fast and accurate identification and typing of BTV we report the development of a complete panel of ‘TaqMan’ fluorescence-probe based quantitative real-time type-specific RT-PCR assays. These real-time RT-PCR assays are considerably more sensitive than the conventional RT-PCR assays previously published for typing BTV RNA [21, 42, 64]. There was also no evidence for cross-amplification of RNA from heterologous serotypes by any of the type-specific assays (Seg-2). However, the very high specificity of real-time RT-PCR primers and probes can lead to false negative results, particularly with strains containing sequence variations in the target gene [65]. Although very recently a set of real-time assays for typing 22 different BTV types have been reported by [66], the combination of type-specific diagnostic assays described here provides a more complete set of tools for rapid and accurate detection and identification of BTV to support surveillance programmes globally. As previously demonstrated for BTV, real-time RT-PCR assays are the most sensitive and reliable methods (that are currently available) for orbivirus detection and typing [67–69]. All of the type-specific assays targeting Seg-2 reported here are highly efficient, doubling amplicon quantity during each round of amplification in the geometric phase of the reaction and can detect similar levels of RNA to other published real-time PCR assays [40, 41]. The large number of BTV isolates that were available for this study from the ORC, have made the wider validation of these assays possible. However, owing to high genetic diversity in these viruses it is very likely that refinement of these assays will be required in future. Eastern and western strains of the same serotype for BTV-1, 2, 9 and 16, were too divergent for a single set of ‘common’ type-specific primers and probe in each case (Table 3). Hence the assays for these serotypes use a duplex format to allow amplification of the more diverse strains (e and w) within these types (S1 Table). Some of the BTV-1 strains from Australia and BTV-4, BTV-12 and BTV-21 isolated from recent outbreaks in India showed poor reactivity with the original pairs of primers and probes (Table 2) hence the primers for these serotypes were upgraded as listed in Table 2. In certain serotypes (BTV-7, -18, -20, -21, -25, -26, -27 and -29) the specificity of amplification was not widely validated as only limited number of isolates, were available for testing, leaving some uncertainty concerning their specificity with more diverse strains of the same types. The diagnostic specificity of the typing assays described here, relates primarily to their inability to detect Seg-2 of non-homologous types, while still detecting all available isolates from the homologous BTV type. If a novel isolate of the virus is identified (e.g. by the virus-species-specific assays) that fails to amplify using the typing assays, it should be sequenced (Seg-2) to provide a basis for further development/refinement of the relevant primers and probes. Similar problems have been addressed with conventional and real-time RT-PCR assays detecting Seg-2 of BTV, in order to maintain their specificity and sensitivity [21].

Previous serological methods have detected multiple serotypes of BTV co-circulating in the endemic regions. The qRT-PCR assays described here have been used to identify several isolates containing mixed BTV types, which could not have been unambiguously typed by VNT.

Recently several novel BTV types (BTV-25–26–27) have been identified primarily by RNA sequence based methods, including RT-PCR assays, and subsequently confirmed as novel serotypes by serological typing methods [13, 14, 17]. Additional virus strains / isolates have been identified that represent two additional putative BTV serotypes. These include BTV-28 from the Middle East (Peter Mertens—Personal communication); and a putative BTV-29 isolated from an alpaca in South Africa [12]. It has been suggested that it would be useful to set absolute values for nucleotide and amino acid variations in Seg-2 and VP2 that can be used to define BTV serotype (Peter Mertens—Personal communication). Current data indicate that viruses within the same serotype can show up to 31.6% nucleotide variation, and 27.4% amino acid variation in Seg-2 and VP2 respectively [11, 18]. Viruses belonging to different serotypes can show up to 71.5% nucleotide identity and 77.8% aa identity, with a minimum of 28.5% nucleotide variation and 22.2% aa variation between serotypes [11, 18]. There is clearly an overlap that makes the identification of clear limits to serotype variation difficult, but it is important to note that this overlap is caused primarily by the existence of different topotypes of Seg-2 and VP2 within individual serotypes. However, if the major different topotypes (for example the eastern and western groups) are considered separately, then the level of variation for isolates within each serotype (also belonging to the same topotype), drops to a maximum of 21.8% nucleotide variation and 13.9% aa variation. This then gives a clear difference of 7.7% nt and 8.3% aa difference between the levels of variation for distinct serotypes and those within the same serotype and topotype. This potentially gives us useful and clear guidance for the identification of the existing serotypes, where both eastern and western strains have already been identified and sequenced. However, if a novel isolate is discovered that fall into this gap, it could represent either a novel serotype that is closely related to, but distinct from, other established serotypes, or it could represent an isolate of an existing serotype, but belonging to a different and distinct major topotype. The novel putative serotypes BTV-28 and -29 show significant sequence variations in Seg-2/VP2 when compared to isolates of previously recognised BTV serotypes, with a level of identity (up to 69.5%), confirming that they represent novel types. Even though the **sero**logical techniques are relatively insensitive and prone to cross-reactions, they remain as a gold-standard for **sero**type identification, particularly for confirmation of novel serotypes. Once identified in this way these new viruses would become a ‘reference strains’, either for a novel serotype, or of an existing serotype but within a new major topotype. This also confirms that we need to identify reference strains for each serotype, within each of the major BTV topotypes.

To summarize, the ‘TaqMan’ probe based real-time RT-PCR based methods described here, represent fast, robust and reliable tools for the detection and typing of BTV. Together with sequencing studies it will help us to better understand the molecular epidemiology (distribution and spread) of these viruses. This system will support investigations of BTV outbreaks and can help to devise strategies for timely implementation of control measures for BTV, particularly vaccination programmes.

## Supporting Information

S1 Table Sequence data used to design real time RT-PCR assays. (DOCX)

S2 Table Specificity of BTV virus-type-specific assays. (DOCX)

S3 Table a—h: Limit of detection of BTV-1, -2, -4, -6, -8, -9, -11 and -16 Seg-2 specific RT-PCR assays with serially diluted recombinant plasmid DNA respectively. (DOCX)

S4 Table Analytical sensitivity and efficiency of type-specific (Seg-2) assays with reference strains of eight European serotypes with serially diluted dsRNA standards. (DOCX)
