# Supplementary material for: Development and Evaluation of Real Time RT-PCR Assays for Detection and Typing of Bluetongue Virus
Source: PLoS One. 2016 Sep 23;11(9):e0163014. doi: 10.1371/journal.pone.0163014 (PMC5035095; doi:10.1371/journal.pone.0163014)
Supplement: S1 Table — (DOCX) [file pone.0163014.s001.docx]

**Table S1: Sequence data used to design real time RT-PCR assays**

| **Virus serotype** | | **Origin** | | | **ORC reference**  **number ^2/^ isolate designation** | **Seg-2 Acc. number** | | | |
| --- | --- | --- | --- | --- | --- | --- | --- | --- | --- |
| **Reference strains ^1^** | | | | | |  | | | |
| BTV-1 | | Republic of South Africa (RSA) | | | RSArrrr/01 | AJ585122 | | | |
| BTV-2 | | Republic of South Africa | | | RSArrrr/02 | AJ585123 | | | |
| BTV-3 | | Republic of South Africa | | | RSArrrr/03 | AJ585124 | | | |
| BTV-4 | | Republic of South Africa | | | RSArrrr/04 | AJ585125 | | | |
| BTV-5 | | Republic of South Africa | | | RSArrrr/05 | AJ585126 | | | |
| BTV-6 | | Republic of South Africa | | | RSArrrr/06 | AJ585127 | | | |
| BTV-7 | | Republic of South Africa | | | RSArrrr/07 | AJ585128 | | | |
| BTV-8 | | Republic of South Africa | | | RSArrrr/08 | AJ585129 | | | |
| BTV-9 | | Republic of South Africa | | | RSArrrr/09 | AJ585130 | | | |
| BTV-10 | | Republic of South Africa | | | RSArrrr/10 | AJ585131 | | | |
| BTV-11 | | Republic of South Africa | | | RSArrrr/11 | AJ585132 | | | |
| BTV-12 | | Republic of South Africa | | | RSArrrr/12 | AJ585133 | | | |
| BTV-13 | | Republic of South Africa | | | RSArrrr/13 | AJ585134 | | | |
| BTV-14 | | Republic of South Africa | | | RSArrrr/14 | AJ585135 | | | |
| BTV-15 | | Republic of South Africa | | | RSArrrr/15 | AJ585136 | | | |
| BTV-16 | | Republic of South Africa | | | RSArrrr/16 | AJ585137 | | | |
| BTV-17 | | Republic of South Africa | | | RSArrrr/17 | AJ585138 | | | |
| BTV-18 | | Republic of South Africa | | | RSArrrr/18 | AJ585139 | | | |
| BTV-19 | | Republic of South Africa | | | RSArrrr/19 | AJ585140 | | | |
| BTV-20 | | Republic of South Africa | | | RSArrrr/20 | AJ585141 | | | |
| BTV-21 | | Republic of South Africa | | | RSArrrr/21 | AJ585142 | | | |
| BTV-22 | | Republic of South Africa | | | RSArrrr/22 | AJ585143 | | | |
| BTV-23 | | Republic of South Africa | | | RSArrrr/23 | AJ585144 | | | |
| BTV-24 | | Republic of South Africa | | | RSArrrr/24 | AJ585145 | | | |
| BTV-25 | | Switzerland | | | SWI2008/01 | EU839840 | | | |
| BTV-26 | | Kuwait | | | KUW2010/02 | HM590642 | | | |
| BTV-27 | | Corsica, France | | | strain 37 | KM200718 | | | |
| BTV-28 | | Middle-east | | | - | - | | | |
| BTV-29 | | Republic of South Africa | | | - | KP196604 | | | |
|  | |  | | |  |  | | | |
| **Sequence data generated during these studies and obtained from international databases** | | | | | | | | | |
| BTV-1 |  | | | |  |  | | | |
|  | Australia | | | | - | X06464 | | | |
|  | Australia | | | | AUS----/01 | AJ585120 | | | |
|  | Australia | | | | AUS1981/01 | AJ585178 | | | |
|  | Australia | | | | - | M21844 | | | |
|  | Australia | | | | AUS- CSIRO156 | M21355 | | | |
|  | Australia | | | | AUS1996/03 | - | | | |
|  | Australia | | | | AUS2003/01 |  | | | |
|  | Australia | | | | AUS2005/02 |  | | | |
|  | Australia | | | | AUS2009/02 |  | | | |
|  | India | | | | IND2001/01 | AJ585115 | | | |
|  | India | | | | IND1992/02 | AJ585112 | | | |
|  | India | | | | IND1999/01 | AJ585114 | | | |
|  | India | | | | IND2003/10 | KP696573 | | | |
|  | India | | | | IND2003/05 | KP696563 | | | |
|  | India | | | | IND2003/04 | KP696553 | | | |
|  | India | | | | IND1985/01 | KP696508 | | | |
|  | India | | | | IND1992/01 | AJ585111 | | | |
|  | India | | | | IND1988/01 | AJ585113 | | | |
|  | Greece | | | | GRE2001/01 | AJ585121 | | | |
|  | Malaysia | | | | MAY1987/01 | AJ585116 | | | |
|  | Peoples Republic of China (PRC) | | | | Y863 | AF135217 | | | |
|  | India | | | | Chennai | AY559061 | | | |
|  | India | | | | Sirsa3 | AY559060 | | | |
|  | RSA vaccine | | | | RSAvvvv/01 | AJ585110 | | | |
|  | RSA | | | | RSArrrr/01 | AJ585122 | | | |
|  | Sudan | | | | SUD1987/01 | AJ585117 | | | |
|  | Cameroon | | | | CAR1982/01 | AJ585119 | | | |
|  | Nigeria | | | | NIG1982/01 | AJ585118 | | | |
|  | Algeria | | | | ALG2006/01 | EU625361 | | | |
|  | Morocco | | | | MOR2006/06 | EU625362 | | | |
|  | USA | | | | USA2004/02 | - | | | |
|  | France | | | | BTV-1/07-01 | FJ437557 | | | |
|  | Oman | | | | OMN2009/01 | - | | | |
|  | Caribbean | | | | Souche215 | - | | | |
|  | Caribbean | | | | Souche108 | - | | | |
|  | Portugal | | | | PT/29058/07 | EU498674 | | | |
|  | France | | | | FRA/07-01 | FJ437557 | | | |
|  | Australia | | | | AUS (west), | X55800 | | | |
|  |  | | | |  |  | | | |
| BTV-2 | | | | | | | | | |
|  | | India | | | M10 | DQ462580 | | | |
|  | | India | | | IND1982/01 | AJ585152 | | | |
|  | | Taiwan | | | Taiwan | AY493687 | | | |
|  | | PRC | | | V440 | AF135218 | | | |
|  | | India | | | IND2003/01 | KP696593 | | | |
|  | | India | | | IND2003/02 | JQ681258 | | | |
|  | | India | | | IND2003/03 | KP696603 | | | |
|  | | Spain | | | SPA2005/01 | AM773697 | | | |
|  | | Portugal (Grandola) | | | strain 26629/PT05 | EF434177 | | | |
|  | | Italy (Sicily) | | | - | DQ191271 | | | |
|  | | USA | | | OnaB | AY855266 | | | |
|  | | USA | | | OnaA | AY855265 | | | |
|  | | RSA | | | 557 SA | AY855264 | | | |
|  | | USA | | | - | M21946 | | | |
|  | | USA | | | - | M21946 | | | |
|  | | RSA vaccine | | | RSAvvvv/02 | AJ585157 | | | |
|  | | RSA | | | RSArrrr/02 | AJ585123 | | | |
|  | | RSA | | | - | AF481096 | | | |
|  | | Nigeria | | | NIG1982/01 | AJ585153 | | | |
|  | | Sudan | | | SUD1985/01 | AJ585155 | | | |
|  | | USA | | | FL99 13406-2 | AY855267 | | | |
|  | | Zimbabwe | | | ZIM2003/01 | - | | | |
|  | | Italy | | | ITL2002/01 | - | | | |
|  | | Campania | | | - | DQ191274 | | | |
|  | | Calabria | | | - | DQ191263 | | | |
|  | | Puglia | | | - | DQ191275 | | | |
|  | | Sardinia | | | - | DQ191273 | | | |
|  | | Sardinia | | | - | DQ191261 | | | |
|  | | Sicily | | | - | DQ191262 | | | |
|  | | Lazio | | | - | DQ191264 | | | |
|  | | Corsica | | | - | AF356601 | | | |
|  | | France | | | FRA2001/03 | AJ585154 | | | |
|  | | Sardinia | | | SAD2001/02 | AJ585162 | | | |
|  | | Italy | | | ITL2002/05 | AJ585158 | | | |
|  | | Italy | | | ITL2002/06 | AJ585159 | | | |
|  | | Italy | | | ITL2002/07 | AJ585160 | | | |
|  | | France (Corsica) | | | - | AF356601 | | | |
|  | | Tunisia | | | TUN2000/01 | AJ585156 | | | |
|  | | Sardinia | | | SAD2001/01 | AJ585161 | | | |
|  | |  | | |  |  | | | |
| BTV-3 | | | | | | | | | |
|  | | RSA | | | RSArrrr/03 | AJ585124 | | | |
|  | | Australia | | | - | X55801 | | | |
|  | | Zimbabwe | | | ZIM2002/01 | AJ585179 | | | |
|  | | France (la Reunion) | | | - | AY485669 | | | |
|  | | USA | | | USA2006/10 |  | | | |
|  | | Australia | | | DPP973 | L42168 | | | |
|  | | PRC | | | V349 | AF135219.2 | | | |
|  | |  | | |  |  | | | |
| BTV-4 | | | | | | | | | |
|  | | Israel | ISR2008/04 | | | | - | | |
|  | | Israel | ISR2008/12 | | | | - | | |
|  | | Greece | GRE2000/07 | | | | - | | |
|  | | RSA vaccine | RSAvvvv/04 | | | | AJ585163 | | |
|  | | RSA vaccine | RSAvvv3/04 | | | | AJ585168 | | |
|  | | Turkey vaccine | TURvvvv/04 | | | | AJ585164 | | |
|  | | Moracco | MOR2004/02 | | | | - | | |
|  | | Spain | SPA2004/01 | | | | - | | |
|  | | Spain | SPA2003/01 | | | | - | | |
|  | | Spain | SPA2003/02 | | | | - | | |
|  | | Spain | SPA2003/03 | | | | - | | |
|  | | Sudan | SUD1983/01 | | | | AJ585166 | | |
|  | | Cyprus | CYP1969/01 | | | | AJ585180 | | |
|  | | RSA | RSArrrr/04 | | | | AJ585125 | | |
|  | | Greece | GRE2000/01 | | | | AJ585167 | | |
|  | | Turkey | TUR1978/01 | | | | AJ585165 | | |
|  | | Israel | ISR2002/06 | | | | - | | |
|  | | Argentina | ARG2002/01 | | | | AJ585169 | | |
|  | | PRC | V413 | | | | AF135220 | | |
|  | | Portugal (Alandroal) | BTV4/22045/PT04 | | | | EF434176 | | |
|  | | Turkey | 1999 | | | | DQ825670 | | |
|  | | RSA | Vaccine strain | | | | AY839948 | | |
|  | | Greece | 1999 | | | | AY839947 | | |
|  | | Greece | 2000 | | | | AY839946 | | |
|  | | Corsica | 2003 | | | | AY839945 | | |
|  | | Italy | Isolate 92069 | | | | DQ191281 | | |
|  | | Sardinia | 2003 | | | | DQ191280 | | |
|  | | Israel | Isolate 3222222 (2001) | | | | DQ191279 | | |
|  | | Israel | Isolate 320226 (2001) | | | | DQ191278 | | |
|  | | Greece | Isolate Mandiko (1999) | | | | DQ191277 | | |
|  | | Greece | 1999 | | | | DQ191276 | | |
|  | | India | IND2014/24 | | | | - | | |
|  | |  |  | | | |  | | |
| BTV-5 | | | | | | | | | |
|  | | RSA | RSArrrr/05 | | | | AJ585126 | | |
|  | | Cameroon | CAR1982/02 | | | | AJ585181 | | |
|  | | Nigeria | NIG1982/03 | | | | AJ585182 | | |
|  | | USA | USA2003/05 | | | | - | | |
|  | | India | IND2010/02 | | | | - | | |
|  | |  |  | | | |  | | |
| BTV-6 | | | | | | | | | |
|  | | RSA | | RSArrrr/06 | | | | AJ585127 | |
|  | | RSA | | Vaccine | | | | GQ506507 | |
|  | | Germany | | 2009 | | | | - | |
|  | | Netherlands | | NET2008/05 | | | | GQ506472 | |
|  | | Netherlands | | NET2008/04 | | | | GU550956 | |
|  | | Netherlands | | NET2008/06 | | | | GU550957 | |
|  | | USA | | USA2006/01 | | | | GQ506536 | |
|  | | RSA | | P635 | | | | GQ506489 | |
|  | | RSA | | from lamb | | | | GQ506517 | |
|  | |  | |  | | | |  | |
| BTV-7 | | | | | | | | | |
|  | | RSA | RSArrrr/07 | | | | | AJ585128 | |
|  | | Australia | AUS2007/01 | | | | | - | |
|  | |  |  | | | | |  | |
| BTV-8 | | | | | | | | | |
|  | | RSA | RSArrrr/08 | | | | | AJ585129 | |
|  | | Kenya | KEN----/01 | | | | | AJ585183 | |
|  | | Netherlands | NET2006/04 | | | | | AM498052 | |
|  | | Belgium | BEL2006/01 | | | | | - | |
|  | | Nigeria | NIG1982/07 | | | | | AJ585184 | |
|  | | RSA | RSA1992/01 | | | | | - | |
|  | | RSA | RSA1987/01 | | | | | - | |
|  | | RSA | RSA1998/01 | | | | | - | |
|  | | Denmark | DEN2007/01 | | | | |  | |
|  | | UK | UKG2007/06 | | | | |  | |
|  | | UK | B411-07 | | | | |  | |
|  | | UK | B463-07-1 | | | | |  | |
|  | | UK | B412-07 | | | | |  | |
|  | | Netherlands | NET2007/01 | | | | | GQ506452 | |
|  | | UK | B252-07 | | | | |  | |
|  | | UK | B356-07-1 | | | | |  | |
|  | | UK | B251/07 | | | | |  | |
|  | | UK | B534-07-1 | | | | |  | |
|  | | Switzerland | A139-07-1 | | | | |  | |
|  | | Netherlands |  | | | | | FJ183375 | |
|  | |  |  | | | | |  | |
| BTV-9 | | | | | | | | | |
|  | | India | IND2005/01 | | | | | KP696633 | |
|  | | India | IND2005/02 | | | | | KP696613 | |
|  | | India | IND2005/03 | | | | | KP696652 | |
|  | | India | IND2004/02 | | | | | KP696613 | |
|  | | India | IND2004/04 | | | | | KP696623 | |
|  | | India (Mahaboobnagar) | 2002 | | | | | DQ872902 | |
|  | | India | K8 | | | | | DQ462581 | |
|  | | Italy (Calabria) | 2001 | | | | | DQ191285 | |
|  | | Italy (Calabria) | 2001 | | | | | DQ191284 | |
|  | | Greece (Rhodes island) | 1998 | | | | | DQ191283 | |
|  | | Indonesia | ISA88 | | | | |  | |
|  | | Indonesia | ISA91 | | | | |  | |
|  | | Australia | DPP836 | | | | | L46686 | |
|  | | Greece | GRE2000/02 | | | | | AJ585171 | |
|  | | Greece (Chalkidiki) | 1999 | | | | |  | |
|  | | Bosnia | BOS2002/02 | | | | | AJ585174 | |
|  | | Serbia | SER2001/01 | | | | | AJ585172 | |
|  | | Bulgaria | BUL1999/01 | | | | | AJ585170 | |
|  | | Turkey | TUR2000/03 | | | | | AJ585175 | |
|  | | Turkey | TUR2000/05 | | | | | AJ585177 | |
|  | | Turkey | TUR2000/04 | | | | | AJ585176 | |
|  | | Italy | ITL2003/01 | | | | | ?? | |
|  | | Italy (Calabria) | - | | | | | DQ191282 | |
|  | |  | RSAvvv1/09 | | | | | AJ585173 | |
|  | |  | RSArrrr/09 | | | | | AJ585130 | |
|  | |  |  | | | | |  | |
| BTV-10 | | | | | | | | | |
|  | | Dominican Republic | DOM1996/01 | | | | |  | |
|  | |  | - | | | | | L29027 | |
|  | | India | IND2005/04 | | | | | - | |
|  | | RSA | RSArrrr/10 | | | | | AJ585131 | |
|  | | USA | USA2002/03 | | | | | - | |
|  | | USA |  | | | | | L29026 | |
|  | | USA |  | | | | | U06786 | |
|  | | USA |  | | | | | U06785 | |
|  | | USA |  | | | | | U06784 | |
|  | | USA |  | | | | | U06783 | |
|  | | USA |  | | | | | U06782 | |
|  | | USA |  | | | | | U06781 | |
|  | | USA |  | | | | | U06780 | |
|  | | USA |  | | | | | M11787 | |
|  | |  |  | | | | |  | |
| BTV-11 | | | | | | | | | |
|  | | Dominican Republic | DOM1996/03 | | | | | - | |
|  | | Zimbabwe | ZIM2003/02 | | | | | - | |
|  | | Zimbabwe | ZIM2003/05 | | | | | - | |
|  | | RSA | RSArrrr/11 | | | | | AJ585132 | |
|  | | USA | - | | | | | M17437 | |
|  | |  |  | | | | |  | |
| BTV-12 | | | | | | | | | |
|  | | Brazil | BRA2002/01 | | | | | - | |
|  | | RSA | RSArrrr/12 | | | | | AJ585133 | |
|  | | Kenya | KEN----/01 | | | | | AJ585185 | |
|  | | Nigeria | NIG1982/09 | | | | | - | |
|  | | Zimbabwe | ZIM2003/04 | | | | | - | |
|  | | India | IND2012/01 | | | | | KC662613 | |
|  | |  |  | | | | |  | |
| BTV-13 | | | | | | | | | |
|  | | Belize | BZE1990/01 | | | | | | - |
|  | | RSA | RSArrrr/13 | | | | | | AJ585134 |
|  | | USA | - | | | | | | D00153 |
|  | | USA | - | | | | | | AY855268 |
|  | | USA | - | | | | | | L11741 |
|  | | USA | - | | | | | | L11874 |
|  | |  |  | | | | | |  |
| BTV-14 | | | | | | | | | |
|  | | Belize | BZE1990/02 | | | | | | - |
|  | | RSA | RSArrrr/14 | | | | | | AJ585135 |
|  | | Cameroon | CAR1982/04 | | | | | | AJ585186 |
|  | | USA | USA2003/03 | | | | | | - |
|  | |  |  | | | | | |  |
| BTV-15 | | | | | | | | | |
|  | | Zimbabwe | ZIM2003/08 | | | | | | - |
|  | | Zimbabwe | ZIM2003/09 | | | | | | - |
|  | | RSA | RSArrrr/15 | | | | | | AJ585136 |
|  | | PRC | V447 | | | | | | AF135221 |
|  | | Israel | ISR2006/11 | | | | | | JF343006 |
|  | |  |  | | | | | |  |
| BTV-16 | | | | | | | | | |
|  | | RSA | RSArrrr/16 | | | | | | AJ585149 |
|  | | Israel | ISR2001/18 | | | | | | AM773710 |
|  | | RSA Vaccine | RSAvvvv/16 | | | | | | AJ585149 |
|  | | Israel | ISR2003/01 | | | | | | - |
|  | | Turkey | TUR2000/01 | | | | | | - |
|  | | Turkey | TUR2000/02 | | | | | | AJ585147 |
|  | | Turkey | TUR2000/10 | | | | | | - |
|  | | Greece | GRE1999/13 | | | | | | AM773709 |
|  | | PRC | - | | | | | | AF135222 |
|  | | Australia | DPP965 | | | | | | L46683 |
|  | | Nigeria | NIG1982/10 | | | | | | AJ585150 |
|  | | Indonesia | ISA1991/01 | | | | | | AJ585151 |
|  | | Italy (Puglia) | - | | | | | | DQ191260 |
|  | | Israel | - | | | | | | DQ191259 |
|  | | France | - | | | | | | AF530067 |
|  | | Israel | ISR2003/01 | | | | | | - |
|  | | Israel | ISR2008/03 | | | | | | - |
|  | | Oman | OMN2009/02 | | | | | | - |
|  | | Sardinia | SAD2004/04 | | | | | | AM773700 |
|  | | Sardinia | SAD2004/03 | | | | | | AM773699 |
|  | | India | IND2014/01 | | | | | | - |
|  | |  |  | | | | | |  |
| BTV-17 | | | | | | | | | |
|  | | RSA | RSArrrr/17 | | | | | | AJ585138 |
|  | | California | - | | | | | | S72158 |
|  | | Canada | - | | | | | | DQ848152 |
|  | | USA | - | | | | | | AY855269 |
|  | | USA | - | | | | | | AY636078 |
|  | | USA | - | | | | | | AY636077 |
|  | | USA | - | | | | | | AY636076 |
|  | | USA | - | | | | | | AY636075 |
|  | | USA | - | | | | | | AY636074 |
|  | | USA | - | | | | | | AY636073 |
|  | | USA | - | | | | | | AY636072 |
|  | | USA | - | | | | | | AY636071 |
|  | | USA | - | | | | | | AF017279 |
|  | | USA | - | | | | | | AF017278 |
|  | | USA | - | | | | | | M17438 |
|  | | Trinidad Tobago | TAT1990/02 | | | | | | - |
|  | |  |  | | | | | |  |
| BTV-18 | | | | | | | | | |
|  | | RSA | RSArrrr/18 | | | | | | AJ585139 |
|  | | Trinidad Tobago | TAT1990/04 | | | | | | - |
|  | |  |  | | | | | |  |
| BTV-19 | | | | | | | | | |
|  | | RSA | RSArrrr/19 | | | | | | AJ585140 |
|  | | USA | USA2003/04 | | | | | | - |
|  | | Dominican Republic | DOM1996/02 | | | | | | - |
|  | | Trinidad Tobago | TAT1990/05 | | | | | | - |
|  | |  |  | | | | | |  |
| BTV-20 | | | | | | | | | |
|  | | RSA | | RSArrrr/20 | | | | | AJ585141 |
|  | | Australia | | AUS1975/01 | | | | | - |
|  | |  | |  | | | | |  |
| BTV-21 | | | | | | | | | |
|  | | RSA | RSArrrr/21 | | | | | | AJ585142 |
|  | | Australia | CSIRO154 | | | | | | L46684 |
|  | | Australia | AUS1979/02 | | | | | | - |
|  | | India | IND2007/09 | | | | | | JF768738 |
|  | |  |  | | | | | |  |
| BTV-22 | | | | | | | | | |
|  | | RSA | RSArrrr/22 | | | | | | AJ585143 |
|  | | Nigeria | NIG1982/11 | | | | | | AJ585187 |
|  | | USA | USA2002/02 | | | | | | - |
|  | | Trinidad Tobago | TAT1990/01 | | | | | | - |
|  | |  |  | | | | | |  |
| BTV-23 | | | | | | | | | |
|  | | India | IND1988/02 | | | | | | JQ771824 |
|  | | India | IND1997/01 | | | | | | AJ585189 |
|  | | India | IND2004/07 | | | | | | KP696671 |
|  | | India | IND2004/08 | | | | | | KP696681 |
|  | | India | IND2004/09 | | | | | | KP696691 |
|  | | RSA | RSArrrr/23 | | | | | | AJ585144 |
|  | | USA | - | | | | | | U04200 |
|  | | Indonesia | ISA1991/02 | | | | | | AJ585188 |
|  | | Australia | DPP0090 | | | | | | L46685 |
|  | | India | Bangalore | | | | | | AY643507 |
|  | | India | Rahuri | | | | | | AY643506 |
|  | | India | Dehradun | | | | | | AY643505 |
|  | |  |  | | | | | |  |
| BTV-24 | | | | | | | | | |
|  | | RSA | RSArrrr/24 | | | | | | AJ585145 |
|  | | USA | USA2007/01 | | | | | | - |
|  | | Israel | ISR2008/05 | | | | | | - |
|  | | India | IND2010/01 | | | | | | KU355785 |
|  | |  |  | | | | | |  |
| BTV-25 | |  |  | | | | | |  |
|  | | Switzerland | SWI2008/01 | | | | | | EU839840 |
|  | |  |  | | | | | |  |
| BTV-26 | |  |  | | | | | |  |
|  | | Kuwait | KUW2010/02 | | | | | | HM590642 |
|  | |  |  | | | | | |  |
| BTV-27 | |  |  | | | | | |  |
|  | | Corsica, France | Isolate 379 | | | | | | KM200718 |
|  | |  |  | | | | | |  |
| BTV-28 | |  |  | | | | | |  |
|  | | Middle-East | - | | | | | | Nomikou et al – unpublished |
|  | |  |  | | | | | |  |
| BTV-29 | |  |  | | | | | |  |
|  | | South Africa | Isolate BT 57/08 | | | | | | KP196604 |
|  | |  |  | | | | | |  |

^1^ Set of 27+1 BTV reference strains [[14](#_ENREF_14), [25](#_ENREF_25)]

^2^ Orbivirus reference collection (ORC), The Pirbright Institute.
