# Supplementary material for: Development and Evaluation of Real Time RT-PCR Assays for Detection and Typing of Bluetongue Virus
Source: PLoS One. 2016 Sep 23;11(9):e0163014. doi: 10.1371/journal.pone.0163014 (PMC5035095; doi:10.1371/journal.pone.0163014)
Supplement: S2 Table — (DOCX) [file pone.0163014.s002.docx]

**Table S2: Specificity of BTV virus-type-specific assays**

| **Virus serotype** | **Origin** | **ORC reference number^1^** | **Average Ct values** |
| --- | --- | --- | --- |
| **Reference strains^2^** |  |  |  |
| BTV-1 | RSA | RSArrrr/01 | 15.32 |
| BTV-2 | RSA | RSArrrr/02 | 16.52 |
| BTV-3 | RSA | RSArrrr/03 | 20.10 |
| BTV-4 | RSA | RSArrrr/04 | 13.97 |
| BTV-5 | RSA | RSArrrr/05 | 17.22 |
| BTV-6 | RSA | RSArrrr/06 | 21.60 |
| BTV-7 | RSA | RSArrrr/07 | 16.32 |
| BTV-8 | RSA | RSArrrr/08 | 17.28 |
| BTV-9 | RSA | RSArrrr/09 | 18.16 |
| BTV-10 | RSA | RSArrrr/10 | 17.66 |
| BTV-11 | RSA | RSArrrr/11 | 20.91 |
| BTV-12 | RSA | RSArrrr/12 | 15.17 |
| BTV-13 | RSA | RSArrrr/13 | 18.92 |
| BTV-14 | RSA | RSArrrr/14 | 21.04 |
| BTV-15 | RSA | RSArrrr/15 | 16.62 |
| BTV-16 | RSA | RSArrrr/16 | 18.26 |
| BTV-17 | RSA | RSArrrr/17 | 17.91 |
| BTV-18 | RSA | RSArrrr/18 | 17.24 |
| BTV-19 | RSA | RSArrrr/19 | 22.80 |
| BTV-20 | RSA | RSArrrr/20 | 15.73 |
| BTV-21 | RSA | RSArrrr/21 | 18.92 |
| BTV-22 | RSA | RSArrrr/22 | 19.53 |
| BTV-23 | RSA | RSArrrr/23 | 18.65 |
| BTV-24 | RSA | RSArrrr/24 | 16.28 |
| BTV-25 | Switzerland | SWI2008/01 | *In silico* |
| BTV-26 |  | KUW2010/02 | 13.73 |
| BTV-27 |  | strain 37 | 11.24 |
| BTV-28 |  | - | *-* |
| BTV-29 |  | - | 15.39 |
| **Other BTV topotypes** |  |  |  |
| BTV-1 | India | IND1992/02 | 17.13 |
| BTV-1 | Morocco | MOR2009/16 | 19.02 |
| BTV-1 | Gibraltar | GIB2007/01 | 18.63 |
| BTV-1 | Morocco | MOR2006/10 | 15.35 |
| BTV-1 | Greece | GRE2001/01 | 19.68 |
| BTV-1 | Malaysia | MAY1987/01 | 16.27 |
| BTV-1 | Nigeria | NIG1982/01 | 19.56 |
| BTV-1 | Cameroon | CAR1982/01 | 18.11 |
| BTV-1 | Australia | AUS1981/03 | 14.94 |
| BTV-2 | Israel | ISR2010/24 | 19.07 |
| BTV-2 | Australia | AUS2008/01 | 17.43 |
| BTV-2 | Martinique | MAR2005/01 | 20.10 |
| BTV-2 | Spain | SPA2005/01 | 14.82 |
| BTV-2 | USA | USA2003/01 | 16.98 |
| BTV-2 | Zimbabwe | ZIM2003/01 | 21.08 |
| BTV-2 | France | FRA2001/05 | 23.74 |
| BTV-2 | Tunisia | TUN2000/02 | 19.77 |
| BTV-2 | Sardinia | SAD2001/01 | 15.81 |
| BTV-2 | Nigeria | NIG1982/02 | 17.37 |
| BTV-2 | India | IND1994/01 | 14.96 |
| BTV-2 | USA | USA2003/01 | 20.31 |
| BTV-3 | India | IND2004/05 | 21.47 |
| BTV-3 | USA | USA2006/10 | 16.68 |
| BTV-3 | France | FRA2003/01 | 15.58 |
| BTV-3 | Zimbabwe | ZIM2002/01 | 21.77 |
| BTV-3 | Trinidad and Tobacco | TAT1990/03 | 19.52 |
| BTV-3 | Nigeria | NIG1982/06 | 19.07 |
| BTV-4 | Bulgaria | BUL2014/01 | 21.33 |
| BTV-4 | Oman | OMN2009/09 | 21.51 |
| BTV-4 | Spain | SPA2003/01 | 13.65 |
| BTV-4 | Argentina | ARG2001/01 | 19.71 |
| BTV-4 | Israel | ISR2001/01 | 16.49 |
| BTV-4 | Greece | GRE1999/01 | 15.87 |
| BTV-4 | Sudan | SUD1983/01 | 16.36 |
| BTV-4 | Egypt | EGY1977/01 | 16.75 |
| BTV-4 | Cyprus | CYP1969/01 | 15.68 |
| BTV-5 | India | IND2012/01 | 23.21 |
| BTV-5 | Israel | ISR2011/01 | 20.64 |
| BTV-5 | USA | USA2003/05 | 12.27 |
| BTV-5 | Cameroon | CAR1982/03 | 21.01 |
| BTV-5 | Nigeria | NIG1982/05 | 18.28 |
| BTV-6 | Greece | GER2008/01 | 18.24 |
| BTV-6 | Netherlands | NET2008/06 | 22.07 |
| BTV-6 | USA | USA2006/01 | 21.54 |
| BTV-7 | Australia | AUS2007/01 | 19.21 |
| BTV-8 | Israel | ISR2009/13 | 17.45 |
| BTV-8 | Oman | OMN2009/03 | 13.73 |
| BTV-8 | France | FRA2006/01 | 19.75 |
| BTV-8 | UK | UKG2007/01 | 14.31 |
| BTV-8 | Netherlands | NET2006/04 | 16.22 |
| BTV-8 | Nigeria | NIG1982/07 | 13.26 |
| BTV-9 | Italy | ITL2003/01 | 18.24 |
| BTV-9 | Bosnia | BOS2002/02 | 22.30 |
| BTV-9 | Martinique | MAR2005/06 | 23.24 |
| BTV-9 | India | IND2003/11 | 18.13 |
| BTV-9 | Libya | LIB2008/06 | 17.80 |
| BTV-9 | Australia | AUS1985/01 | 19.84 |
| BTV-10 | India | IND2004/01 | 15.64 |
| BTV-10 | Martinique | MAR2005/02 | 14.31 |
| BTV-10 | Zimbabwe | ZIM2003/06 | 18.67 |
| BTV-10 | Dominican Republic | DOM1996/01 | 16.59 |
| BTV-10 | USA | USA1980/02 | 16.91 |
| BTV-11 | USA | USA 1976/01 | 18.59 |
| BTV-11 | Nigeria | NIG1982/08 | 23.76 |
| BTV-11 | Dominican Republic | DOM1996/03 | 23.09 |
| BTV-11 | Zimbabwe | ZIM2003/02 | 13.81 |
| BTV-12 | Israel | ISR2010/26 | 13.91 |
| BTV-12 | Zimbabwe | ZIM2003/04 | 13.99 |
| BTV-12 | Nigeria | NIG1982/09 | 14.23 |
| BTV-12 | India | IND2010/02 | 16.99 |
| BTV-12 | Brazil | BRA2002/01 | 13.76 |
| BTV-13 | Martinique | MAR2009/02 | 18.09 |
| BTV-13 | Belize | BZE1990/01 | 14.87 |
| BTV-13 | USA | USA1977/01 | 14.86 |
| BTV-14 | USA | USA2003/03 | 17.76 |
| BTV-14 | Belize | BZE1990/02 | 21.97 |
| BTV-14 | Cameroon | CAR1982/04 | 14.52 |
| BTV-15 | Israel | ISR2006/11 | 14.65 |
| BTV-15 | Zimbabwe | ZIM2003/08 | 20.89 |
| BTV-15 | Australia | AUS1982/01 | 15.60 |
| BTV-16 | India | IND2007/01 | 17.72 |
| BTV-16 | Israel | ISR2009/01 | 14.55 |
| BTV-16 | Greece | GRE2009/01 | 18.05 |
| BTV-16 | Indonesia | ISA1991/01 | 12.89 |
| BTV-16 | Oman | OMN2009/02 | 31.54 |
| BTV-17 | Martinique | MAR2005/04 | 16.95 |
| BTV-17 | Trinidad and Tobacco | TAT1990/02 | 11.90 |
| BTV-17 | USA | USA2006/03 | 12.65 |
| BTV-18 | Martinique | MAR2005/07 | 12.96 |
| BTV-18 | Trinidad and Tobacco | TAT1990/04 | 13.68 |
| BTV-19 | Trinidad and Tobacco | TAT1990/05 | 14.05 |
| BTV-19 | Dominican Republic | DOM1996/02 | 16.70 |
| BTV-19 | USA | USA2003/04 | 15.67 |
| BTV-20 | Australia | AUS1975/01 | 20.61 |
| BTV-21 | India | IND2007/09 | 22.75 |
| BTV-21 | Australia | AUS1979/02 | 21.90 |
| BTV-22 | Nigeria | NIG1982/11 | 20.54 |
| BTV-22 | Trinidad and Tobacco | TAT1990/01 | 12.89 |
| BTV-22 | USA | USA2002/02 | 15.87 |
| BTV-22 | Martinique | MAR2005/05 | 12.90 |
| BTV-23 | India | IND1988/02 | 14.64 |
| BTV-23 | Nepal | NEP2007/01 | 16.88 |
| BTV-23 | Australia | AUS1982/02 | 18.53 |
| BTV-24 | India | IND2010/01 | 19.09 |
| BTV-24 | Israel | ISR2008/05 | 12.76 |
| BTV-24 | USA | USA2007/01 | 16.79 |
| BTV-24 | Martinique | MAR2005/03 | 11.76 |
|  |  |  |  |
| EEV-1 | RSA | RSA1967/03 | No Ct |
| EEV-2 | RSA | RSA1971/06 | No Ct |
| EEV-3 | RSA | RSA1974/06 | No Ct |
| EEV-4 | RSA | RSA1976/03 | No Ct |
| EEV-5 | RSA | RSA1976/06 | No Ct |
| EEV-6 | RSA | RSA1991/03 | No Ct |
| EEV-7 | RSA | - | No Ct |
|  |  |  |  |
| PHSV | Peru | PER1997/01 | No Ct |
|  |  |  |  |
| EHDV-1 | USA | USA1955/01[W] | No Ct |
| EHDV-1 | Australia | AUS1995/02[E] | No Ct |
| EHDV-1 | Nigeria | NIG1967/01 | No Ct |
| EHDV-2 | Japan | JAP1959/01[E] | No Ct |
| EHDV-2 | Australia | AUS1979/05 | No Ct |
| EHDV-2 | Canada | CAN1962/01[W] | No Ct |
| EHDV-2 | Japan | JAP----/08 | No Ct |
| EHDV-4 | Nigeria | NIG1968/01[W] | No Ct |
| EHDV-5 | Australia | AUS1977/01[E] | No Ct |
| EHDV-6 | Australia | AUS1981/07[E] | No Ct |
| EHDV-6 | Bahrain | BAR1983/01[W] | No Ct |
| EHDV-7 | Australia | AUS1981/06[E] | No Ct |
| EHDV-8 | Australia | AUS1982/06[E] | No Ct |
|  |  |  |  |
| AHSV-1 | RSA | RSArah1/03 | No Ct |
| AHSV-2 | RSA | RSArah2/03 | No Ct |
| AHSV-3 | RSA | RSArah3/03 | No Ct |
| AHSV-4 | RSA | RSArah4/03 | No Ct |
| AHSV-5 | RSA | RSArah5/03 | No Ct |
| AHSV-6 | RSA | RSArah6/03 | No Ct |
| AHSV-7 | RSA | KENrah7/03 | No Ct |
| AHSV-8 | RSA | RSArah8/03 | No Ct |
| AHSV-9 | RSA | PAKrah9/03 | No Ct |
|  |  |  |  |
| Palyam virus (PALV) | Sudan | SUD1982/03 | No Ct |
| Eubenangee virus (EUBV) | Australia | AUS1978/03 | No Ct |
| Andasibe virus | Madagascar | MAD1979/01 | No Ct |
| Chobar Gorge virus | Nepal | NEP1970/01 | No Ct |
| Japanaut virus | Papua New Guinea | PNG1965/01 | No Ct |
| Matucare virus | Bolivia | BOL1963/01 | No Ct |
| Tembe virus | Brazil | BRA1963/01 | No Ct |
| Tracambe virus | Brazil | BRA1985/01 | No Ct |
| Wallal virus | Australia | AUS1978/09 | No Ct |
| Warrego virus | Australia | AUS2010/01 | No Ct |
| Corriparta virus | Australia | AUS1979/03 | No Ct |
| Ieri virus | Port of Spain, Trinidad | TAT1955/01 | No Ct |
| Palyam virus | Australia | AUS1991/01 | No Ct |
|  |  |  |  |
| *C. sonorensis* | - | - | No Ct |
| Uninfected BHK cells | - | - | No Ct |
| Uninfected Vero cells | - | - | No Ct |
| Uninfected KC cells | - | - | No Ct |
| Uninfected sheep blood | - | - | No Ct |

^1^ Orbivirus reference collection (ORC), The Pirbright Institute.

^2^ Set of 29 BTV reference strains.

Representatives of different serotypes and topotypes from different *Orbivirus* species were tested to confirm the specificity of assays for BTV dsRNA. Further details on these isolates can be obtained from ORC <http://www.reoviridae.org/dsRNA_virus_proteins/ReoID/viruses-at-iah.htm> . EEV = Equine encephalosis virus; PHSV = Peruvian horse sickness virus; EHDV = Epizootic haemorrhagic disease virus; BTV = Bluetongue virus; AHSV = African horse sickness virus; PALV = Palyam virus. RSA = Republic of South Africa.
