# Supplementary material for: Development and Evaluation of Real Time RT-PCR Assays for Detection and Typing of Bluetongue Virus
Source: PLoS One. 2016 Sep 23;11(9):e0163014. doi: 10.1371/journal.pone.0163014 (PMC5035095; doi:10.1371/journal.pone.0163014)
Supplement: S3 Table — (DOCX) [file pone.0163014.s003.docx]

**Supplementary data**

Table S3a – S3h: Limit of detection of BTV-1, -2, -4, -6, -8, -9, -11 and -16 Seg-2 specific RT-PCR assays with serially diluted recombinant plasmid DNA respectively.

Table S3a: Limit of detection of Seg-2 BTV-1 specific RT-PCR assay with serially diluted recombinant plasmid DNA.

| **Recombinant plasmid designation** | **Mean Ct value for**  **BTV-1 (RSArrrr/01)** | **Number of copies/PCR** |
| --- | --- | --- |
| pBTV-1 10^-2^ | 9.9 | 3.088x10^8^ |
| pBTV-1 10^-3^ | 13.6 | 3.088x10^7^ |
| pBTV-1 10^-4^ | 16.6 | 3.088x10^6^ |
| pBTV-1 10^-5^ | 20.5 | 3.088x10^5^ |
| pBTV-1 10^-6^ | 23.8 | 3.088x10^4^ |
| pBTV-1 10^-7^ | 27.1 | 3.088x10^3^ |
| pBTV-1 10^-8^ | 30.6 | 309 |
| pBTV-1 10^-9^ | 33.5 | 31 |
| pBTV-1 10^-10^ | 36.4 | 3.1 |
| pBTV-1 10^-10^ D1/2 | >40 | - |
| pBTV-1 10^-10^ D1/4 | >40 | - |
|  |  |  |

The limit of detection of BTV-1 Seg-2 assay is 3 copies of plasmid/PCR

Table S3b: Limit of detection of Seg-2 BTV-2 specific RT-PCR assay with serially diluted recombinant plasmid DNA.

| **Recombinant plasmid designation** | **Mean Ct value for**  **BTV-2 (RSArrrr/02)** | **Number of copies/PCR** |
| --- | --- | --- |
| pBTV-2 10^-3^ | 12.2 | 1.261x10^8^ |
| pBTV-2 10^-4^ | 15.8 | 1.261x10^7^ |
| pBTV-2 10^-5^ | 19.1 | 1.261x10^6^ |
| pBTV-2 10^-6^ | 22.6 | 1.261x10^5^ |
| pBTV-2 10^-7^ | 26.1 | 1.261x10^4^ |
| pBTV-2 10^-8^ | 29.4 | 1.261x10^3^ |
| pBTV-2 10^-9^ | 32.1 | 126 |
| pBTV-2 10^-10^ | 35.8 | 12.6 |
| pBTV-2 10^-10^ D1/2 | 36.7 | 6.3 |
| pBTV-2 10^-10^ D1/4 | 37.1 | 3.1 |
| pBTV-2 10^-10^ D1/8 | >40 | - |
|  |  |  |

The limit of detection of BTV-2 Seg-2 assay is 3 copies of plasmid/PCR

Table S3c: Limit of detection of Seg-2 BTV-4 specific RT-PCR assay with serially diluted recombinant plasmid DNA.

| **Recombinant plasmid designation** | **Mean Ct value for**  **BTV-4 (RSArrrr/04)** | **Number of copies/PCR** |
| --- | --- | --- |
| pBTV-4 10^-3^ | 11.3 | 2.181x10^8^ |
| pBTV-4 10^-4^ | 14.6 | 2.181x10^7^ |
| pBTV-4 10^-5^ | 18.9 | 2.181x10^6^ |
| pBTV-4 10^-6^ | 22.4 | 2.181x10^5^ |
| pBTV-4 10^-7^ | 26.2 | 2.181x10^4^ |
| pBTV-4 10^-8^ | 30.1 | 2.181x10^3^ |
| pBTV-4 10^-9^ | 33.2 | 218.1 |
| pBTV-4 10^-10^ | 36.4 | 21.81 |
| pBTV-4 10^-10^ D1/2 | 37.9 | 10.9 |
| pBTV-4 10^-10^ D1/4 | >40 | - |
|  |  |  |

The limit of detection of BTV-4 Seg-2 assay is 11 copies of plasmid/PCR

Table S3d: Limit of detection of Seg-2 BTV-6 specific RT-PCR assay with serially diluted recombinant plasmid DNA.

| **Recombinant plasmid designation** | **Mean Ct value for**  **BTV-6 (RSArrrr/06)** | **Number of copies/PCR** |
| --- | --- | --- |
| pBTV-6 10^-2^ | 9.2 | 1.197x10^9^ |
| pBTV-6 10^-3^ | 12.4 | 1.197x10^8^ |
| pBTV-6 10^-4^ | 15.4 | 1.197x10^7^ |
| pBTV-6 10^-5^ | 18.4 | 1.197x10^6^ |
| pBTV-6 10^-6^ | 22.3 | 1.197x10^5^ |
| pBTV-6 10^-7^ | 25.2 | 1.197x10^4^ |
| pBTV-6 10^-8^ | 28.6 | 1.197x10^3^ |
| pBTV-6 10^-9^ | 32.4 | 119.7 |
| pBTV-6 10^-10^ | 35.1 | 11.97 |
| pBTV-6 10^-10^ D1/2 | 36.3 | 5.98 |
| pBTV-6 10^-10^ D1/4 | 37.2 | 3 |
| pBTV-6 10^-10^ D1/8 | >40 | - |
|  |  |  |

The limit of detection of BTV-6 Seg-2 assay is 3 copies of plasmid/PCR

Table S3e: Limit of detection of Seg-2 BTV-8 specific RT-PCR assay with serially diluted recombinant plasmid DNA.

| **Recombinant plasmid designation** | **Mean Ct value for**  **BTV-8 (RSArrrr/08)** | **Number of copies/PCR** |
| --- | --- | --- |
| pBTV-8 10^-2^ | 8.0 | 1.002x10^9^ |
| pBTV-8 10^-3^ | 11.2 | 1.002x10^8^ |
| pBTV-8 10^-4^ | 14.3 | 1.002x10^7^ |
| pBTV-8 10^-5^ | 17.4 | 1.002x10^6^ |
| pBTV-8 10^-6^ | 21.1 | 1.002x10^5^ |
| pBTV-8 10^-7^ | 24.2 | 1.002x10^4^ |
| pBTV-8 10^-8^ | 27.4 | 1.002x10^3^ |
| pBTV-8 10^-9^ | 30.8 | 100.2 |
| pBTV-8 10^-10^ | 34.1 | 10 |
| pBTV-8 10^-10^ D1/2 | 39.0 | 5 |
| pBTV-8 10^-10^ D1/4 | >40 | - |
|  |  |  |

The limit of detection of BTV-8 Seg-2 assay is 5 copies of plasmid/PCR

Table S3f: Limit of detection of Seg-2 BTV-9 specific RT-PCR assay with serially diluted recombinant plasmid DNA.

| **Recombinant plasmid designation** | **Mean Ct value for**  **BTV-9 (RSArrrr/09)** | **Number of copies/PCR** |
| --- | --- | --- |
| pBTV-9 10^-2^ | 7.6 | 8.703x10^8^ |
| pBTV-9 10^-3^ | 11.2 | 8.703x10^7^ |
| pBTV-9 10^-4^ | 13.9 | 8.703x10^6^ |
| pBTV-9 10^-5^ | 18.8 | 8.703x10^5^ |
| pBTV-9 10^-6^ | 22.1 | 8.703x10^4^ |
| pBTV-9 10^-7^ | 25.4 | 8.703x10^3^ |
| pBTV-9 10^-8^ | 28.7 | 870.3 |
| pBTV-9 10^-9^ | 31.4 | 87 |
| pBTV-9 10^-10^ | 33.6 | 8.7 |
| pBTV-9 10^-10^ D1/2 | 35.1 | 4.4 |
| pBTV-9 10^-10^ D1/4 | 37.5 | 2.2 |
| pBTV-9 10^-10^ D1/8 | >40 | - |

The limit of detection of BTV-9 Seg-2 assay is 2 copies of plasmid/PCR

Table S3g: Limit of detection of Seg-2 BTV-11 specific RT-PCR assay with serially diluted recombinant plasmid DNA.

| **Recombinant plasmid designation** | **Mean Ct value for**  **BTV-11 (RSArrrr/11)** | **Number of copies/PCR** |
| --- | --- | --- |
| pBTV-11 10^-2^ | 8.7 | 1.003x10^9^ |
| pBTV-11 10^-3^ | 12.3 | 1.003x10^8^ |
| pBTV-11 10^-4^ | 15.4 | 1.003x10^7^ |
| pBTV-11 10^-5^ | 19.2 | 1.003x10^6^ |
| pBTV-11 10^-6^ | 21.9 | 1.003x10^5^ |
| pBTV-11 10^-7^ | 24.8 | 1.003x10^4^ |
| pBTV-11 10^-8^ | 29.3 | 1.003x10^3^ |
| pBTV-11 10^-9^ | 32.7 | 100.3 |
| pBTV-11 10^-10^ | 35.4 | 10 |
| pBTV-11 10^-10^ D1/2 | 37.2 | 5 |
| pBTV-11 10^-10^ D1/4 | >40 | - |
| pBTV-11 10^-10^ D1/8 |  |  |

The limit of detection of BTV-11 Seg-2 assay is 5 copies of plasmid/PCR

Table S3h: Limit of detection of Seg-2 BTV-16 specific RT-PCR assay with serially diluted recombinant plasmid DNA.

| **Recombinant plasmid designation** | **Mean Ct value for**  **BTV-16 (RSArrrr/16)** | **Number of copies/PCR** |
| --- | --- | --- |
| pBTV-16 10^-2^ | 8.2 | 1.164x10^9^ |
| pBTV-16 10^-3^ | 11.7 | 1.164x10^8^ |
| pBTV-16 10^-4^ | 14.8 | 1.164x10^7^ |
| pBTV-16 10^-5^ | 18.2 | 1.164x10^6^ |
| pBTV-16 10^-6^ | 21.6 | 1.164x10^5^ |
| pBTV-16 10^-7^ | 25.2 | 1.164x10^4^ |
| pBTV-16 10^-8^ | 28.2 | 1.164x10^3^ |
| pBTV-16 10^-9^ | 31.6 | 116.4 |
| pBTV-16 10^-10^ | 35.9 | 11.64 |
| pBTV-16 10^-10^ D1/2 | 37.6 | 5.8 |
| pBTV-16 10^-10^ D1/4 | >40 | - |
|  |  |  |

The limit of detection of BTV-16 Seg-2 assay is 6 copies of plasmid/PCR
