# Supplementary material for: Development and Evaluation of Real Time RT-PCR Assays for Detection and Typing of Bluetongue Virus
Source: PLoS One. 2016 Sep 23;11(9):e0163014. doi: 10.1371/journal.pone.0163014 (PMC5035095; doi:10.1371/journal.pone.0163014)
Supplement: S4 Table — (DOCX) [file pone.0163014.s004.docx]

**Supplementary data**

Table S4: Analytical sensitivity and efficiency of type-specific (Seg-2) assays with reference strains of eight European serotypes with serially diluted dsRNA standards.

| **Virus isolate serotype** | **Virus isolate designation** | **Number of molecules detected** | **Efficiency (%)** | **RSq** | **Slope** |
| --- | --- | --- | --- | --- | --- |
| **BTV-1** | RSArrrr/01 | 3 | 99 | 0.9988 | -3.339507 |
| **BTV-2** | RSArrrr/02 | 3 | 100 | 0.9990 | -3.303852 |
| **BTV-4** | RSArrrr/04 | 11 | 101 | 0.9998 | -3.237641 |
| **BTV-6** | RSArrrr/06 | 3 | 101 | 0.9995 | -3.280395 |
| **BTV-8** | RSArrrr/08 | 5 | 102 | 0.9997 | -3.259781 |
| **BTV-9** | RSArrrr/09 | 2 | 100 | 0.9953 | -3.312978 |
| **BTV-11** | RSArrrr/11 | 5 | 97 | 0.9991 | -3.385489 |
| **BTV-16** | RSArrrr/16 | 6 | 95 | 0.9982 | -3.444927 |
